# Rigid Bronchoscopy: A Life-Saving Intervention in the Removal of Foreign Body in Adults at a Busy Tertiary Care Unit

**DOI:** 10.7759/cureus.9662

**Published:** 2020-08-11

**Authors:** Misauq Mazcuri, Tanveer Ahmad, Khalil A Shaikh, Ambreen Abid, Shagufta Nasreen, Nazish Sikander

**Affiliations:** 1 Thoracic Surgery, Jinnah Postgraduate Medical Centre, Karachi, PAK

**Keywords:** rigid bronchoscopy, foreign body, secondary bronchus, scarf pin

## Abstract

Introduction

Foreign body (FB) aspiration is a potentially lethal emergency and is not uncommon in adults. Rigid bronchoscopy (RB) is a useful procedure for the extraction of these FBs, and it has a high success rate. The aim of this study was to document the clinical presentation of FB aspirations and management outcomes of non-vegetative FB extraction using RB as a therapeutic modality.

Method

This prospective interventional study was conducted in the Department of Thoracic Surgery, Jinnah Postgraduate Medical Centre (JPMC), Karachi from May 2019 to April 2020. Patients of both genders of ages 12 years or above, presenting with FB aspiration were included. RB was performed in all patients.

Results

Chest radiograph (CXR) identified FBs in all 60 patients, 51 of whom were females and nine males. In 24 (40%) patients, a CT scan was performed to accurately localize the FB. Left bronchus was the most common location of the FB (n=39; 65%). Scarf pin was the most common type (n=45; 75%) of FB, followed by sewing needle (n=7; 11.7%), safety pin (n=5; 8.3%), and tire repair needle (n=3; 5%). In 53 (88.3%) patients, RB was successful in retrieving the FB. Thoracotomy was performed in the remaining seven patients due to inaccessibility. One (1.7%) patient died due to the rupture of the thoracic aortic aneurysm.

Conclusion

Accidental aspiration of pins and needles can be fatal in adults. RB is a life-saving modality for safely removing these FBs. However, thoracotomy should be used as a life-saving procedure in cases of FBs affecting secondary bronchi or beyond.

## Introduction

Aspiration of foreign body (FB) is a surgical emergency. It may turn out to be fatal and accounts for almost three-fourths of all accidents in children. Aspiration of FB in adults, although not frequently encountered, is not an uncommon finding [1]. Rigid bronchoscopy (RB) and flexible bronchoscopy (FLB) are useful modalities for the extraction of FBs [2,3]. Patients with impaction of FB in the upper respiratory tract usually present with choking of sudden onset, cough, shortness of breath, or difficulty in breathing, which may even be severe enough to result in acute respiratory failure [2,4]. If the acute event goes unnoticed, it may lead to chronic complications such as recurrent pneumonia, hemoptysis, pneumothorax, bronchiectasis, lung abscesses, and perforation of a viscus. Although there is not enough data in the literature on FB aspiration in adults, reports indicate that 0.16-0.33% of bronchoscopies performed in adults are for FB extraction [4].

Delayed diagnosis and peripherally located FBs may result in failed removal attempts [5]. Chest radiograph (CXR) and CT remain essential in determining the characteristics of radio-opaque FBs, including their shape, size, location, and position [1]. Radiopaque FBs can be directly visualized on CXR, whereas radiolucent FBs may present with signs of indirect airway obstruction (atelectasis, unilateral hyperinflation, or localized bronchiectasis) [2].

For extraction of an airway FB in adults, RB and FLB are being widely utilized. Both procedures have their own advantages, and FLB has the benefit of avoidance of general anaesthesia, easy-to-use instruments, and widespread availability. However, the failure rate of FLB is not negligible, especially when the diagnosis is delayed and in cases of peripherally located FBs [5-7]. Difficulty in maintaining airway patency can be a challenge, and complete cooperation of the patient is essential for the procedure. These factors somewhat reduce the efficacy of FLB in FB retrieval. On the other hand, RB for FB retrieval has shown a high success rate [7]. Other than the availability of general anesthesia, there is no other limitation regarding the use of RB in the extraction of FBs.

There have been occasional case reports of adult FB aspiration from Pakistan [8,9]. There is still a dearth of published material on this interventional study in Pakistan, and to the best of our knowledge, only one recent study has been published on this subject [10]. Our study aims to assess the presentation of FB aspirations and the management outcomes of FB extraction with RB.

## Materials and methods

A prospective, interventional study was conducted in the Department of Thoracic Surgery, Jinnah Postgraduate Medical Centre (JPMC), Karachi. The duration of this study was one year, from May 1, 2019, to April 30, 2020. The study was conducted after obtaining ethical approval from the institutional review board of JPMC. Informed consent was obtained from all study participants.

All patients of either gender aged 12 years and above, presenting to the emergency department due to FB aspiration were included. Initial workup included CXRs [both posteroanterior (PA) and lateral views] to determine the type, size, shape, location, and position of the FBs. In cases where secondary bronchus was involved and RB was unsuccessful, a CT chest was done to localize the FB.

After the localization of FB within the airway, RB under general anesthesia was performed. A CXR was performed immediately before the procedure to confirm the position of the FB. All procedures were performed by the same surgeon with a 7.5-mm ventilating rigid bronchoscope. A ventilating rigid bronchoscope has an internal diameter of 6-8 mm and a length of 41-43 cm and is equipped with a ventilating port to insufflate oxygen during the procedure. Alligator Retrieval Forceps® (Cook Group Incorporated, Bloomington, IN) were used to grip and extract the FBs.

All relevant clinical and demographic details of the patients were systematically recorded in pre-devised data form. Procedural details like the number of attempts required, need for a thoracotomy, and any complications encountered were recorded. Statistical analysis was performed using SPSS Statistics for Windows version 22.0 (IBM, Armonk, NY). Continuous variables were presented as mean and standard deviations (SD). Categorical variables were presented as frequencies and percentages. A Chi-square test was applied for correlation.

## Results

Sixty patients were included in this study. There were 51 (85%) females and 9 (15%) males. The mean age of the patients was 19.4 ± 4.6 years (range: 12-28 years). Most of the patients belonged to the age group of 16-20 years (n=24; 40%); there were 14 (23.3%) patients in the age group of 12-15 years, 12 (20%) patients were between 21-25 years old, and 10 (16.6%) patients were in the age group of 26-30 years. The median duration of FB aspiration was 12 hours. Most of the patients (n=21; 35%) presented with 11-15 hours of aspiration.

**Figure 1:**
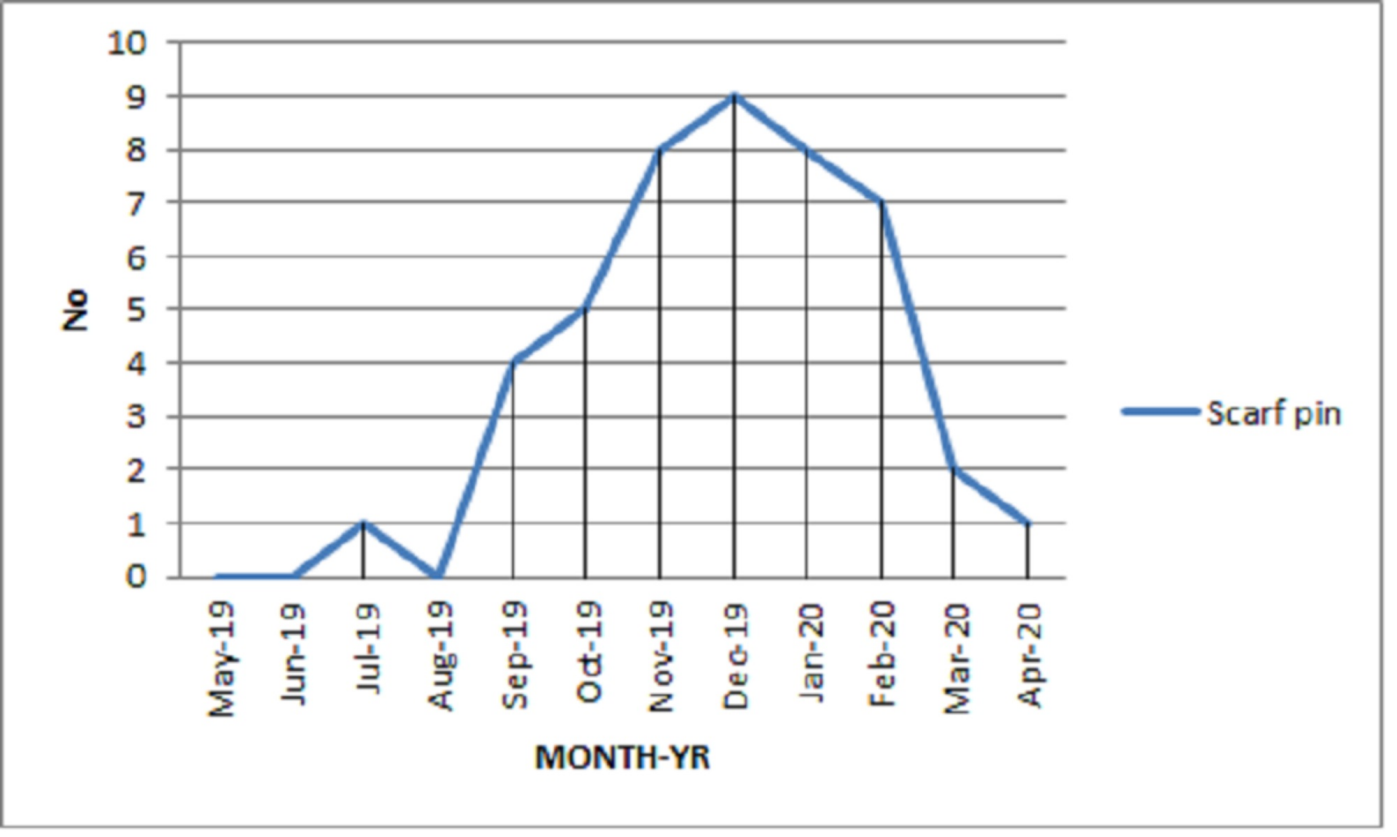
Number of scarf-pin inhalations presenting in different months

CXR was used to find the location and type of FB in all patients. In 24 (40%) patients, a CT scan was performed to further help with localization of the FB, all being cases in which the FB impacted secondary bronchi or beyond (p<0.005) and presenting after 24 hours of aspiration (p=0.005). This group also included 13 (21.7%) cases where FB could not be visualized during the first attempt of RB (p<0.005). There were four types of FBs identified. Scarf pin was the most common (n=45; 75%), followed by sewing needle (n=7; 11.7%), safety pin (n=5; 8.3%), and tire repair needle (n=3; 5%). Most of the scarf-pin inhalations were seen between November and February. Figure 1 represents a graph showing scarf-pin presentations in different months.

**Figure 2:**
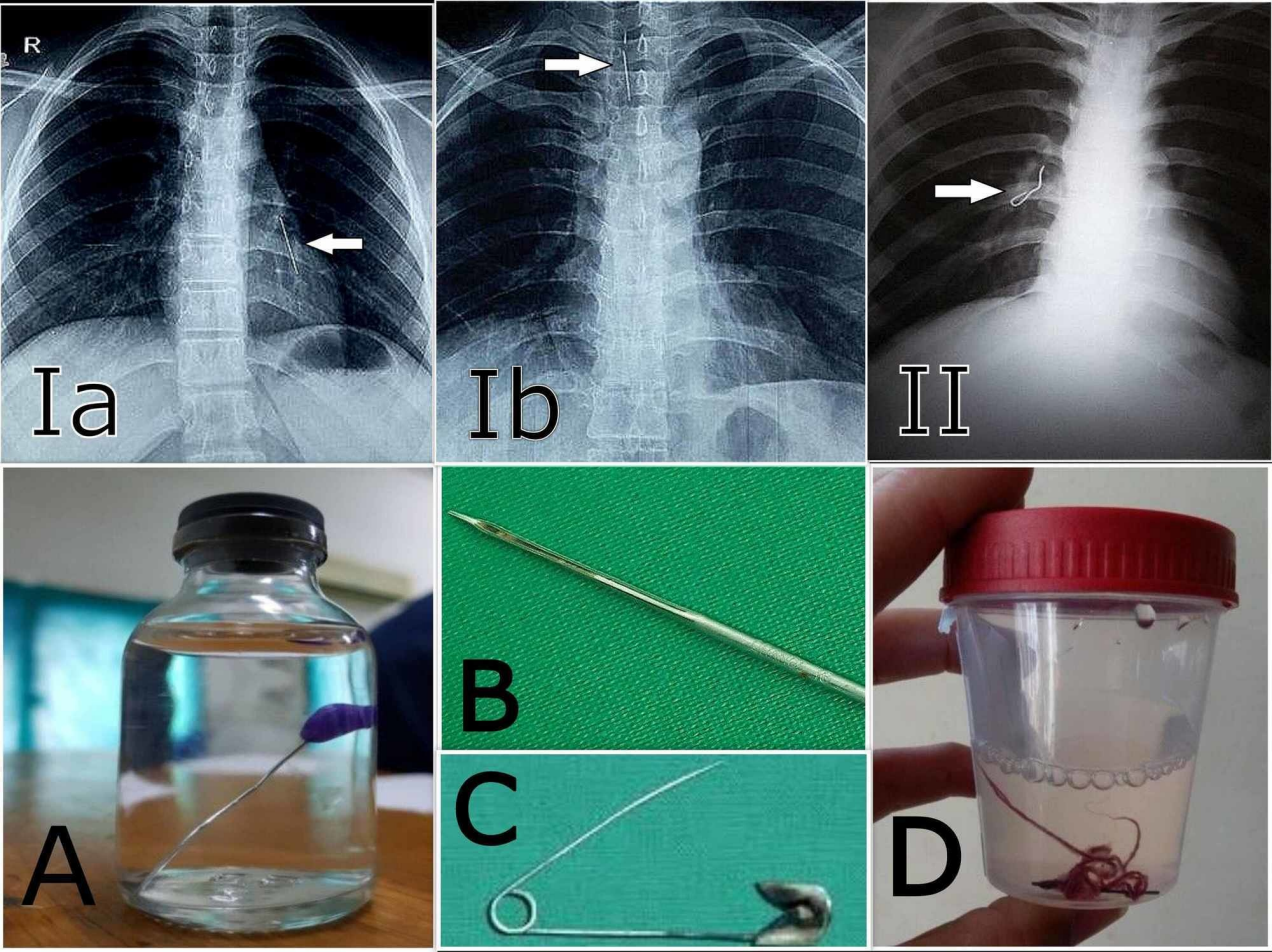
Chest X-rays showing locations of foreign bodies (1a-II); images of different types of foreign bodies removed (A-D) Ia: scarf pin in the left airway; Ib: scarf pin in the trachea; II: sewing needle in the right airway; A: scarf pin; B: tire repair needle; C: safety pin; D: sewing needle

Left bronchus was the most common location of FB (n=39; 65%). In 23 (40%) patients, the FB involved secondary bronchi, of which 16 (69.5%) patients’ FB was removed successfully with RB, while seven (30.4%) patients required thoracotomy as FBs could not be visualized. Figure 2 shows CXRs displaying the location of FBs along with different types of FBs removed by RB.

**Figure 3:**
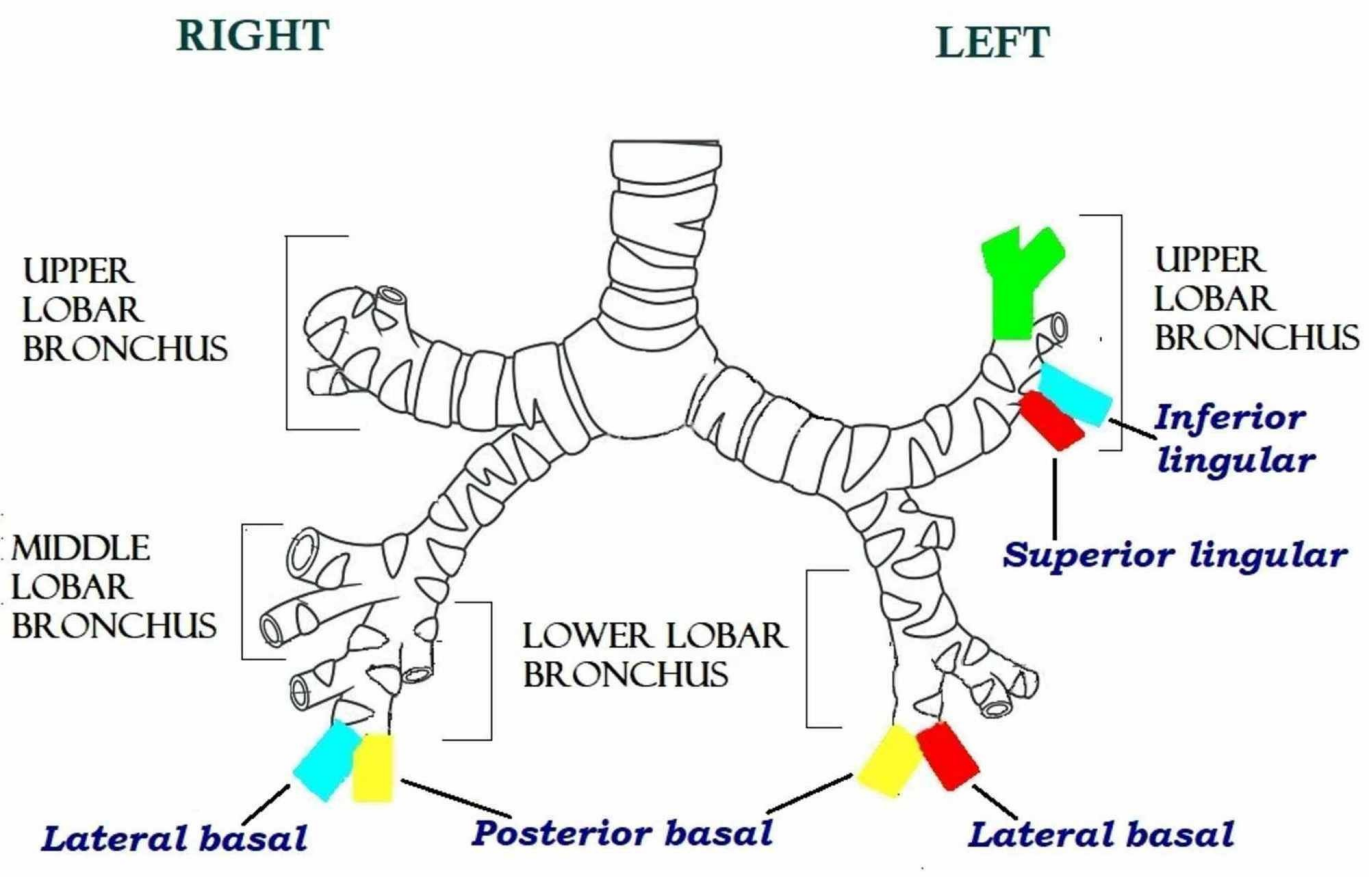
Pictorial representation of the bronchial system from where the foreign bodies were extracted

RB was performed in all patients. It was successful in 53 (88.3%) patients, whereas seven (11.7%) patients required thoracotomy. Figure 3 shows a pictorial representation of the bronchial system from where FBs were extracted via thoracotomy and bronchotomy [11].

Patients who underwent thoracotomy required a CT scan to identify the site of FB (p=0.001), with most of them presenting after 24 hours of accidental aspiration (p=0.002) along with the impaction of FB in secondary bronchi or beyond (p<0.005). One of the patients who underwent thoracotomy died due to the rupture of the thoracic aortic aneurysm leading to massive hemoptysis on the fifth postoperative day. The mean duration of hospital stay was 2.83 ± 2.95 days (range: 1-16 days). The clinical data of patients and the characteristics of the procedure and its outcomes are given in Table 1.

**Table 1:** Clinical data of patients and characteristics of the procedure and its outcome (n=60) SD: standard deviation

Patient data and procedure characteristics	Frequency, n (%)
Positive history of aspiration	60 (100%)
Presenting complaint	
Cough	29 (48.3%)
Hemoptysis	16 (26.7%)
Asymptomatic	16 (26.7%)
Chest pain	8 (13.3%)
Dyspnea	3 (5%)
Initial radiological investigation	
Chest radiograph	60 (100%)
Radiological Investigation for definitive localization	
Chest radiograph	36 (60%)
Computed tomography	24 (40%)
Localization of foreign body during the procedure (n=60)	
Within the trachea	3 (5%)
Left airway	39 (65%)
Left main bronchus only	23 (58.9%)
Left secondary bronchus + main bronchus	16 (41.01%)
Left superior lobar	6 (37.5%)
Left inferior lobar	10 (62.5%)
Right airway	18 (30%)
Right main bronchus only	11 (61.1%)
Right secondary bronchus + main bronchus	7 (38.8%)
Right middle lobar	3 (42.8%)
Right inferior lobar	4 (57.14%)
Characteristics of the procedure	
Success of the procedure	
Successful extraction in the first attempt	40 (66.7%)
Successful extraction in the second attempt	13 (21.7%)
Conversion to thoracotomy	7 (11.7%)
Duration of hospital stay, days	
Mean ± SD	2.83 ± 2.95
1 day	38 (63.3%)
2 days	7 (11.7%)
3 days	5 (8.3%)
4 days	3 (5%)
More than 5 days	7 (11.7%)
Post-procedure symptoms	
Cough	36 (60%)
None	15 (25%)
Hemoptysis	7 (11.7%)
Chest pain	5 (8.3%)
Fever	1 (1.7%)
Post-procedure complications	
Hemothorax/pneumothorax (post-thoracotomy)	7 (11.7%)
Atelectasis with ruptured aortic aneurysm with massive hemoptysis	1 (1.7%)
Outcome	
Discharge	59 (98.3%)
Mortality	1 (1.7%)

## Discussion

FB aspiration in adults is most commonly seen in males, with the majority of patients having certain factors that make them susceptible to aspiration, such as neurological deficit causing problems in swallowing, or altered mental status, neuromuscular disease, and intoxication [2,4]. However, recent studies have shown an increase in the number of FB aspiration among females [12-17].

In this study, the majority of patients were females, and the most common FB aspirated was scarf pin. Metallic scarf pin aspiration has been frequently reported from Muslim countries, especially in the Middle East [12-17]. This finding has a close association with religious and cultural values as most women in Muslim regions of the world wear headscarves and, hence, they frequently come into contact with metallic scarf pins. We came across an incidental finding that 32 (71.1%) cases of scarf-pin inhalations presented in the winter season. It may indicate certain associations with social customs with more inclination towards complete head coverage modified according to seasonal fashion. Among males, safety pins, sewing needles, and tire repair needle were identified as aspirated FBs, and these findings mostly pertained to their respective occupations. None of the patients had risk factors or conditions mentioned earlier [2,4].

In this study, the most common clinical presentation of FB aspiration was cough. This finding is consistent with that of most of the reports in the literature [5,12,18]. In contrast, an Egyptian study has reported that only 7% of the 83 cases of metallic hairpin aspirations presented with cough [17]. The triad of cough, hemoptysis, and dyspnea was not seen [1]. According to the literature, 10-20% of patients with metallic pin inhalations are asymptomatic [13]; in our study, 16 (26.7%) patients were asymptomatic.

In the present study, CXRs were helpful in the diagnosis and localization of FBs. CXR was repeated before shifting the patient to the procedure room irrespective of the RB attempt. In the study by Rizk et al., repeated CXR was performed only prior to the second attempt of RB [17]. In 24 (40%) patients, a CT scan was done to accurately localize the site of FB. The first attempt at retrieval with RB was successful in 40 patients; Among the remaining 20, a second attempt with RB proved successful in 13 patients, after CT scans were ordered (p<0.005). Out of the 24 cases requiring CT scan, 23 patients had involvement of secondary bronchi (p<0.005). Although CXR is always recommended as the initial radiological procedure in the diagnosis of FBs, its diagnostic yield is not high, and varying results have been reported in the literature. In a retrospective analysis of FBs extracted bronchoscopically, CXR was normal for 25% of cases [5]. In a study by Zaghba et al., CXR was remarkable for all cases [16]. In our study, patients presenting after 24 hours of inhalation had FBs lodged in secondary bronchi or beyond (p=0.003).

Almost two-thirds of the cases had FB impaction in the left bronchus in our study. Theoretically, the right bronchus is more prone to FB impaction due to its more vertical course as compared to the left [19]. In children, both bronchi are equally prone to FB impaction [20,21]. Strangely, half of the adult FBs were found lodged in the left bronchus, and the remaining were located in the trachea [3,4,13,18]. According to various authors, the Bernoulli phenomenon explains the dislodgement of FBs into the left bronchus during coughing, speaking, and sneezing because of the stronger negative pressure in the narrow left bronchus as compared to the right [17,22]. Some authors have identified the right bronchus as the predominant site of FB impaction [2,5,12,23].

In previous studies, it has been proposed that the beaded end of the pin, due to its heavy nature, tends to face downward towards the distal bronchus whereas the pointed end faces upwards. However, we did not encounter any significant findings that were consistent with this theory [17]. The pointed end can damage the airway during removal; hence, the rigid bronchoscope is an ideal tool as it provides protection from the pointed end of the needle, as the pin is delivered into the hollow tube of the rigid bronchoscope prior to removal from the airway [4].

In our study, RB was performed in all patients and was successful in the first attempt in 40 patients (66.7%); in the remaining 20, a second attempt with RB was made. The second attempt was successful in another 13 patients (21.7%). Conversion to thoracotomy was indicated in seven (11.7%) patients, all of them having FB lodged deeper in the secondary bronchus or beyond (p=0.003) and presenting after 24 hours of inhalation (p=0.002). In an Egyptian report, the success rate of RB was 80% in the first attempt, and repeat RB was performed in 20% of cases, out of which 6% had to be converted to thoracotomy with no mortality [17]. Cobanoglu et al. have reported that RB was successful in 71% of cases, with 14% requiring thoracotomy; 10% required flexible bronchoscopy and 5% were managed with laryngoscopy [12].

In an Indian study, the success rate of RB was 86% in adults, with the remaining being managed with bronchotomy; no mortality was reported [23]. Our study had one postoperative death, which occurred on the fifth day after the procedure. The patient presented with hemoptysis and had a history of accidental aspiration of scarf pin nine months earlier. A CT scan revealed atelectasis, pneumonia, and thoracic aortic aneurysm in addition to a scarf needle lodged next to the aortic wall. RB did not visualize an FB and it was decided to proceed with thoracotomy after the patient was stabilized. A scarf pin was removed successfully in consultation with cardiac surgeons. Early recovery from thoracotomy was uneventful, but on the fifth postoperative day, she had an episode of massive hemoptysis and could not be revived. In another study, the success rate of scarf-pin extraction from the airway through RB was 48% and thoracotomy was needed in only 8% of cases. One death was reported from the RB group secondary to massive endobronchial hemorrhage during FB extraction; we did not have any mortality during RB [22].

In a study by Arshad et al., patients were discharged after 24 hours after RB [10]. In our study, patients were discharged within 12 hours of the RB procedure, after a chest X-ray ruling out any complications. Patients were called for follow-ups after one week. Thoracotomy patients were discharged after the removal of tube thoracostomy and once the lung was fully expanded; they were followed up for wound care and stitch removal.

Our study highlights that accidental FB aspiration, although uncommon in adults, must be diagnosed and managed early to avoid distal displacement in order to avoid thoracotomy. RB remains the gold standard treatment modality for FB extraction owing to its better airway control, maintenance of airway, better management of asphyxiating FBs, and its ability to provide protection against sharp FBs, along with other advantages [4]. RB requires skill and can be a tricky procedure for new surgical residents; hence, RBs should be ideally performed only in dedicated thoracic centers where thoracotomy can be readily undertaken if required.

## Conclusions

Accidental aspiration of pins and needles can be fatal in adults. RB has a high success rate in the extraction of airway FBs and is an important life-saving surgical skill that should be mastered by all thoracic surgeons. However, failure to visualize FBs, intraoperative and postoperative complications, and the urgent need for thoracotomy cannot be overlooked. Early diagnosis and prompt attempt at the extraction of sharp FBs are paramount for the success of the procedure and avoidance of complications, which can be fatal at times.
